# Pathophysiological Relevance of Renal Medullary Conditions on the Behaviour of Red Cells From Patients With Sickle Cell Anaemia

**DOI:** 10.3389/fphys.2021.653545

**Published:** 2021-03-19

**Authors:** David C.-Y. Lu, Rasiqh Wadud, Anke Hannemann, David C. Rees, John N. Brewin, John Stanley Gibson

**Affiliations:** ^1^Department of Veterinary Medicine, University of Cambridge, Cambridge, United Kingdom; ^2^Department of Paediatric Haematology, King’s College Hospital, London, United Kingdom

**Keywords:** HbS polymerisation, sickling, phosphatidylserine, renal medulla, hypoxia, pH, hypertonicity, urea

## Abstract

Red cells from patients with sickle cell anaemia (SCA) contain the abnormal haemoglobin HbS. Under hypoxic conditions, HbS polymerises and causes red cell sickling, a rise in intracellular Ca^2+^ and exposure of phosphatidylserine (PS). These changes make sickle cells sticky and liable to lodge in the microvasculature, and so reduce their lifespan. The aim of the present work was to investigate how the peculiar conditions found in the renal medulla – hypoxia, acidosis, lactate, hypertonicity and high levels of urea – affect red cell behaviour. Results show that the first four conditions all increased sickling and PS exposure. The presence of urea at levels found in a healthy medulla during antidiuresis, however, markedly reduced sickling and PS exposure and would therefore protect against red cell adherence. Loss of the ability to concentrate urine, which occurs in sickle cell nephropathy would obviate this protective effect and may therefore contribute to pathogenesis.

## Introduction

Red cells from patients with sickle cell anaemia (SCA; HbSS genotype) contain a mutated haemoglobin, HbS, which replaces the normal adult HbA (Bunn and Forget, 1986). HbS results from a point mutation of the DNA causing replacement of glutamic acid with valine at the sixth residue of the Hb β chain. This substitution allows neighbouring molecules of HbS to polymerise upon deoxygenation, forming long, rigid concatenations of Hb, which distort the normal red cell shape from biconcave disc to the distinctive sickle shapes together with other bizarre forms. The result is a sticky red cell with poor rheological properties and a propensity to lodge in areas of the microvasculature (Hebbel, 1991).

HbS polymerisation underlies all of the deleterious sequelae experienced by SCA patients. Complications, however, are multiple (Steinberg, 1999; Rees et al., 2010). Reduced red cell life span results in anaemia and ischaemic problems including pain, osteonecrosis, stroke, acute chest syndrome, proliferative retinopathy and others, one of which is nephropathy.

A further feature of sickle cells is a pronounced exposure of the aminophospholipid phosphatidylserine (PS; Kuypers, 1998; de Jong et al., 2002). PS in normal red cells is restricted to the inner leaflet of the membrane bilayer, but it is externalised in a high, but variable, proportion of sickle cells. This is important because PS exposure is prothrombotic and makes cells attractive to phagocytes and activated endothelial cells (Hebbel, 1991). It may therefore affect the lifespan of red cells and contribute to both the anaemia and ischaemic complications of SCA. Three transport systems are implicated in the distribution of PS (Haest, 2003): an aminophospholipid transferase (or flippase), which uses ATP to move PS from the outer to the inner leaflet of the bilayer; a Ca^2+^-activated scramblase, which rapidly shuttles PS across the membrane in both directions; and a floppase, which transports PS outwards.

Loss of PS asymmetry requires inhibition of the flippase together with activation of the scramblase, both of which can occur at low micromolar concentrations of intracellular Ca^2+^ (Bitbol et al., 1987; Woon et al., 1999; Weiss et al., 2012). In normal red cells, intracellular Ca^2+^ is kept at low levels, around 30 nM – at some five orders of magnitude less than the plasma concentration of Ca^2+^ of 1.1 mM – through a combination of meagre membrane permeability to Ca^2+^ and a high capacity plasma membrane Ca^2+^ pump (or PMCA). Sickle cells demonstrate both a reduced PMCA activity and a higher passive Ca^2+^ permeability, particularly following deoxygenation (Rhoda et al., 1990; Etzion et al., 1992) and the sickling shape change which opens a non-specific cation conductance sometimes referred to as P_sickle_ (Joiner, 1993; Gibson and Ellory, 2002; Lew and Bookchin, 2005). The rise in Ca^2+^ may partially explain PS exposure.

Sickle cell anaemia patients also have a markedly increased incidence of nephropathy with about a third progressing to a dependence on renal dialysis or transplantation (Powars et al., 2005; Serjeant et al., 2007). Inability to concentrate urine – hyposthenuria – occurs at an early age with necrosis and fibrosis of the renal medulla commonly observed and may progress to chronic renal failure. The detailed pathogenesis underlying these manifestations remains unclear; however, the unique environment found in the renal medulla is implicated (Wigfall et al., 2000; Becton et al., 2010; Aygun et al., 2011; Elmariah et al., 2014). This tissue has a particularly low blood flow, less than 1% of the total renal circulation. It is, in addition, markedly hypoxic (an O_2_ partial pressure of about 15 mmHg is sometimes quoted, Brezis et al., 1994; Liss et al., 1997; Zhang et al., 2014) and acidic due to anaerobic metabolism, with accumulation of lactate at about 15 mM (Thomas, 2000). Furthermore, during maximal antidiuresis, the medulla is also hypertonic with accumulation of salt (up to 300 mM NaCl) and urea (up to 600 mM), with a total osmolality in healthy adults of about 1,200 mOsm.kg^−1^. Hypoxia, acidosis, and hypertonicity all stimulate HbS polymerisation. It is therefore likely that they will encourage sickling and PS exposure and these factors coupled with a sluggish rate of blood flow may serve to promote adhesion and death of sickle cells, and increase ischaemia and its sequelae in the renal medulla.

The effects on red cells of hypertonicity have been studied extensively by the group of Lang et al. (2002), albeit mainly on normal (HbA) red cells. Stimulation of PS exposure was found to involve several pathways: cation channels permeable to Ca^2+^ (Lang et al., 2004), stimulation of sphingomyelinase and ceramide production (Lang et al., 2004), possibly *via* stimulation of cyclooxygenase (Lang et al., 2005). Lang’s group also showed a protective effect of urea on PS exposure in normal red cells and platelets (Lang et al., 2004; Gatidis et al., 2010), probably *via* inhibition of sphingomyelinase.

In this report, we investigated how these factors – hypoxia, low pH, lactate, hypertonicity, and urea – alter red cell sickling and PS exposure, and hence affect the lifespan of these cells. Unlike previous groups, our study has controlled oxygen tension using levels appropriate to those found in the renal medulla. We found that, as expected, these conditions are associated with increased sickling and PS exposure. We also found, however, that high urea levels acted to ameliorate these changes. A mechanism is proposed, together with a postulated pathophysiological significance.

## Materials and Methods

### Chemicals

Fluorescein isothiocyanate-conjugated lactadherin (LA-FITC) came from Haematologic Technologies Inc. (Essex Junction, VT, United States), supplied by Cambridge Bioscience (Cambridge, United Kingdom). 4-(2-hydroxyethyl)-1-piperazineethanesulfonic acid (HEPES) and 3-(*N*-morpholino) propanesulfonic acid (MOPS) came from Calbiochem (Merck, Darmstadt, Germany). All other chemicals were supplied by Sigma-Aldrich Co. (Poole, Dorset, United Kingdom).

### Sample Collection and Handling

Consented blood samples were taken from patients homozygous for SCA, HbSS genotype, using the anticoagulant EDTA, which is standard for clinical samples. The study was approved by the National Research Ethics Committee (reference 16/LO/1309). For some experiments, once routine haematological assays had been completed, discarded, and anonymised blood was used. All research was conducted with ethical approval and in accordance with the Helsinki Declaration of 1975, as revised in 2008.

### Solutions and Red Cell Preparation

The standard salines buffered with HEPES or MOPS and comprised (in mM): NaCl 145, KCl 5, MgCl_2_ 0.15, inosine 10, and HEPES or MOPS 10, (pH 7.4 at 37°C; 290 ± 5 mOsm.kg^−1^). Inosine protects against the initial reduction in ATP levels when red cells are warmed to 37°C after being stored on ice (Tiffert et al., 1984). The identity of the buffer had no effect on results. Where different pHs were used, saline pH was adjusted using NaOH or HCl. For hypertonicity experiments, tonicity was altered through addition of NaCl or sucrose. Exposed PS was labelled with LA-FITC (16 nM) at pH 7.4 at room temperature (RT) in the presence of 1 mM vanadate (LA-FITC binding buffer). Vanadate was only added after incubation of red cells, just prior to PS measurement. To prepare red cells, whole blood was washed four times in saline (pH 7.4 at RT) to remove plasma and buffy coat. Red cells were stored on ice until required. Haematocrit (Hct) was measured using Drabkin’s reagent.

### Measurement of Sickling

To promote sickling and lipid scrambling, red cells were incubated using three different conditions – hypoxia (30 and 0 mmHg oxygen tension), altered pH (6.0–8.0), and hypertonicity – in gently rotating Eschweiler tonometers at 1% Hct at 37°C for up to 80 min. To measure sickling, as defined in Al Balushi et al. (2019), red cell aliquots were then fixed using 0.3% glutaraldehyde, under the same conditions as incubation – these low levels of glutaraldehyde were chosen because of their minimal effect on saline osmolality. For each condition, 100 cells were assessed and sickling given as a percentage.

### Measurement of Externalised Phosphatidylserine Using LA-FITC

To measure PS, red cells were pelleted and resuspended in buffer containing 1 mM vanadate, diluted in LA-FITC binding buffer at 0.01% Hct, and incubated for 15 min in the dark at RT. Red cells were next pelleted (10 s at 16,100 g), washed once in LK-HBS, resuspended and kept on ice in the dark until flow cytometry analysis. LA-FITC was detected in the FL1 channel of a BD Accuri C6 flow cytometer using logarithmic gain. The positive fluorescent gate was set using red cells unlabelled with LA-FITC. For each measurement, 10,000 events were gated. PS positive cells were defined as all events falling within the preset FSC, SSC, and positive fluorescent gates. See Cytlak et al. (2013) for detailed Methodology.

### Statistics

**Figure 1:**
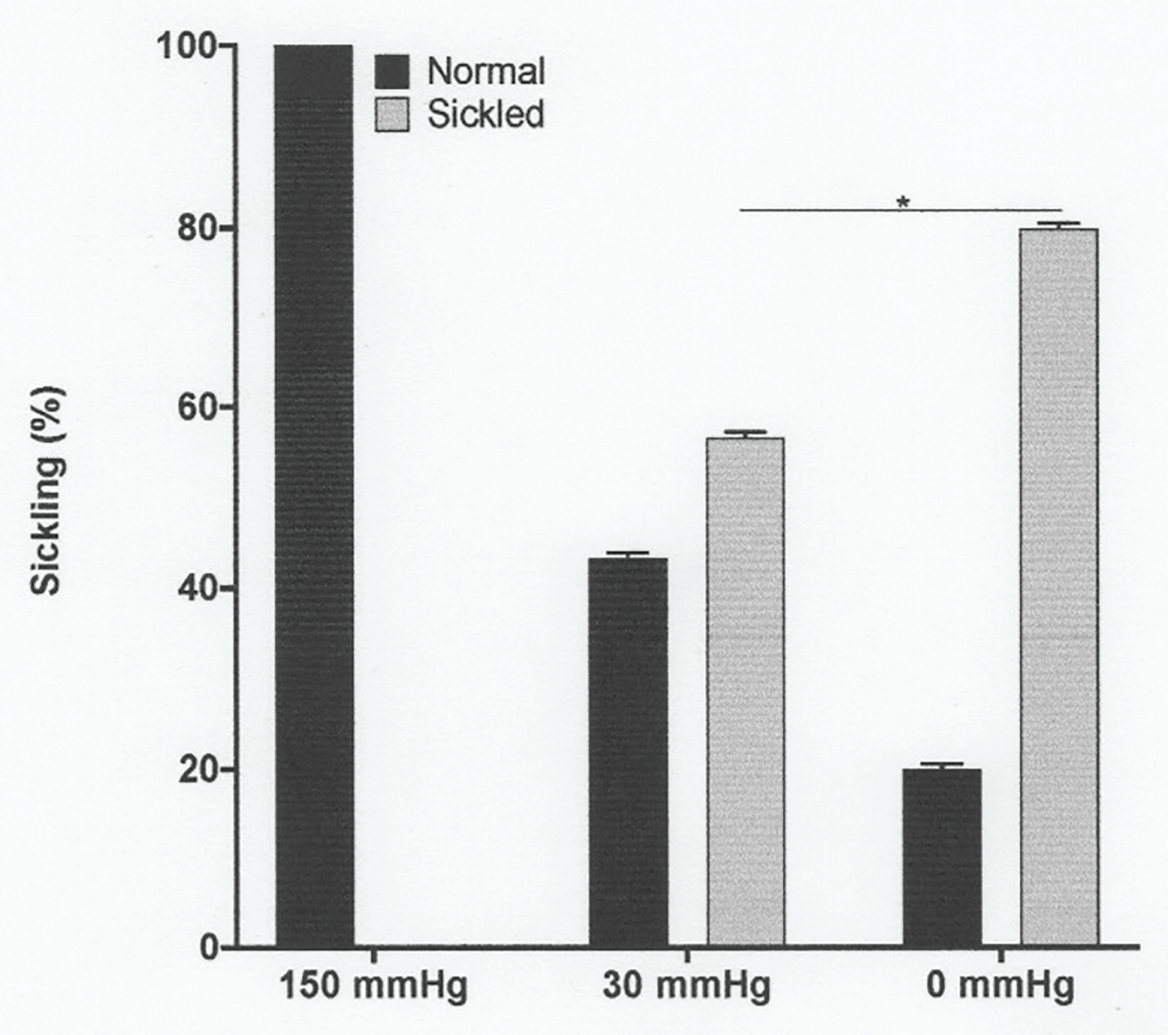
The effect of oxygen tension on sickling of red cells from patients with sickle cell anaemia (SCA). Red cells (1% haematocrit, Hct) were incubated in Eschweiler tonometers at 37°C and pH 7.4 for 15 min and equilibrated with warm humidified gas at three different oxygen tension (150, 30, and 0 mmHg oxygen – air replaced with nitrogen). Red cell aliquots were then removed and fixed using 0.3% glutaraldehyde, whilst maintaining the same oxygen tension present during their incubation. Histograms represent means ± SEM, *n* = 3. ^*^p < 0.05.

**Figure 2:**
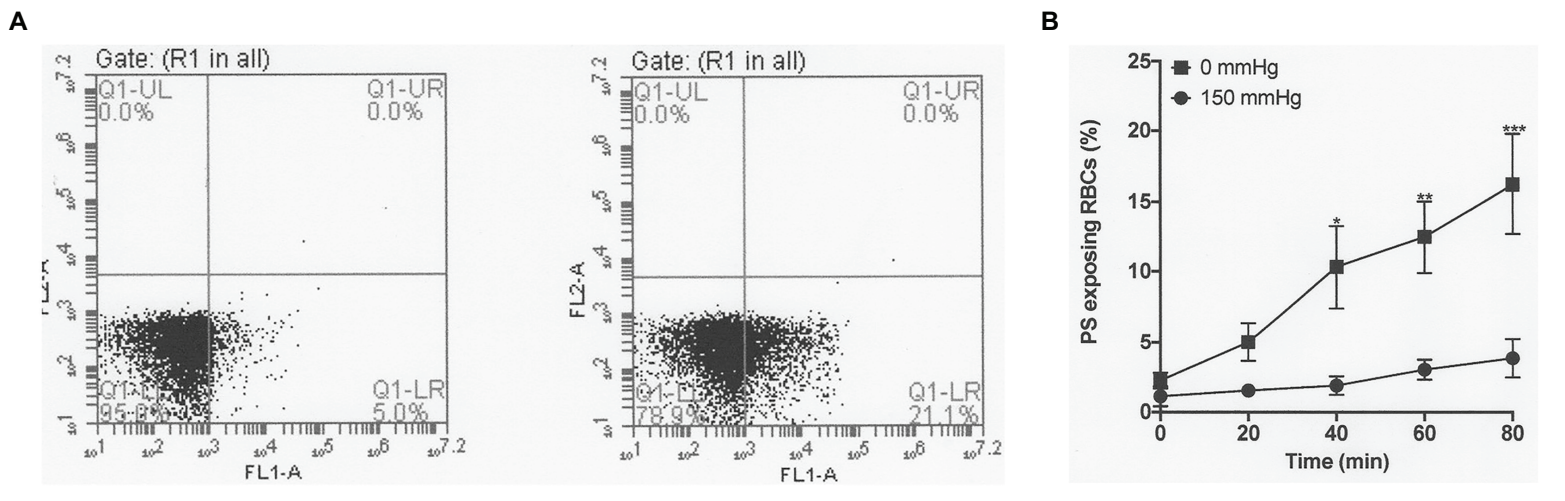
The effect of oxygen tension on phosphatidylserine (PS) exposure in red cells from patients with SCA. Red cells (1% Hct) were incubated in Eschweiler tonometers at 37°C and pH 7.4 and an osmolality of 290 mOsm.kg^−1^ for up to 80 min under fully oxygenated (150 mmHg oxygen) or fully deoxygenated (0 mmHg oxygen) conditions. At the time intervals indicated, red cell aliquots were removed and PS exposure measured using fluorescently-labelled lactadherin (LA-FITC), as described in the Methods. **(A)** Representative FACS result from a single experiment after 80 min, in which PS positive red cells increased from 5.0% when incubated at 150 mmHg oxygen (left panel) to 21.1% at 0 (right panel). **(B)** Averaged data from three separate experiments. Symbols represent means ± SEM, *n* = 3. ^*^p < 0.05; ^**^p < 0.01; and ^***^p < 0.001.

**Figure 3:**
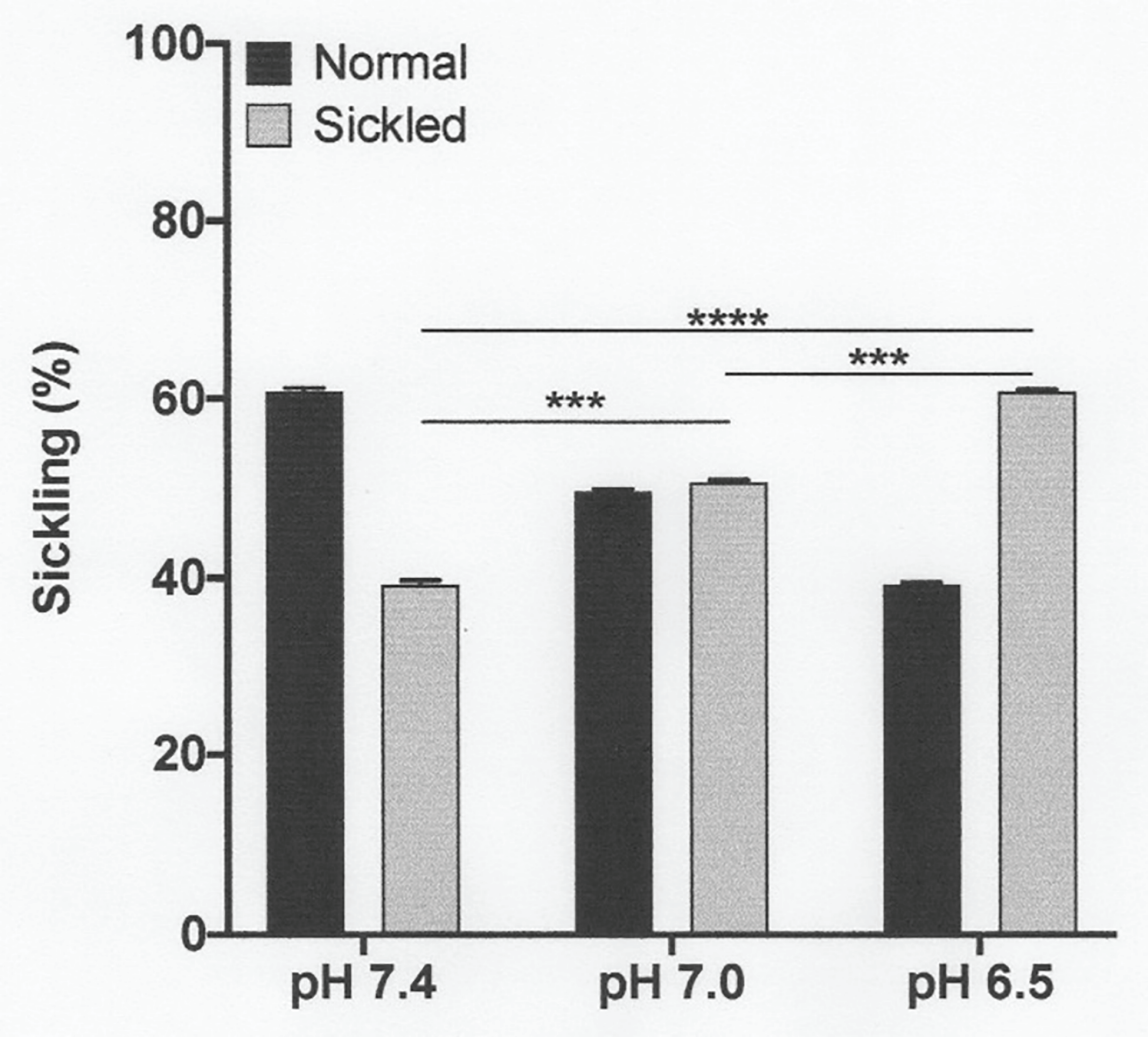
Effect of pH on sickling of deoxygenated red cells from patients with SCA. Red cells (1% haematocrit, Hct) were incubated in Eschweiler tonometers at 37oC and an osmolality of 290 mOsm.kg-1 for 15 min under fully deoxygenated conditions (0 mmHg oxygen) at three different extracellular pH values, pH 7.4, 7.0, and 6.5. Red cell aliquots were then removed and fixed using 0.3% glutaraldehyde whilst maintaining the same pH present during their incubation. Histograms represent means ± SEM, *n* = 3. ^***^p < 0.001; and ^****^p < 0.0001, comparing red cells in the absence of urea with those in its presence.

**Figure 4:**
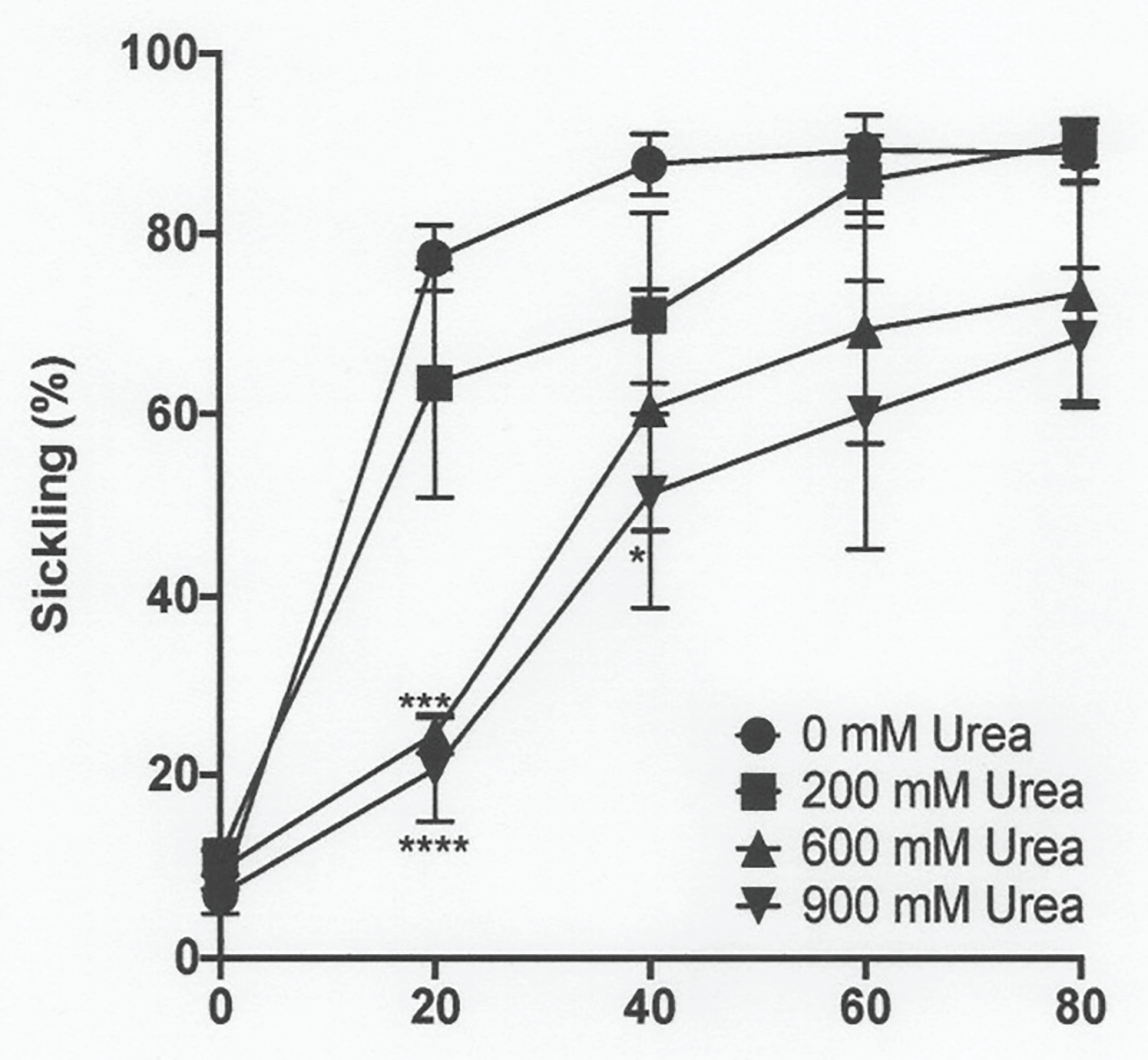
The effect of urea on sickling of deoxygenated red cells from patients with SCA. Red cells (1% haematocrit, Hct) were incubated in Eschweiler tonometers at 37oC, pH 7.4 and an osmolality of 290 mOsm.kg-1 for up to 80 min under fully deoxygenated conditions (0 mmHg oxygen) in the absence of urea (0 mM urea) or at three different urea concentrations (200, 600, and 900 mM). Red cell aliquots were then removed and fixed using 0.3% glutaraldehyde whilst maintaining the same urea concentration present during their incubation. Symbols represent means ± SEM, *n* = 4. ^*^p < 0.05; ^***^p < 0.001; and ^****^p < 0.0001, comparing red cells in the absence of urea with those in its presence.

**Figure 5:**
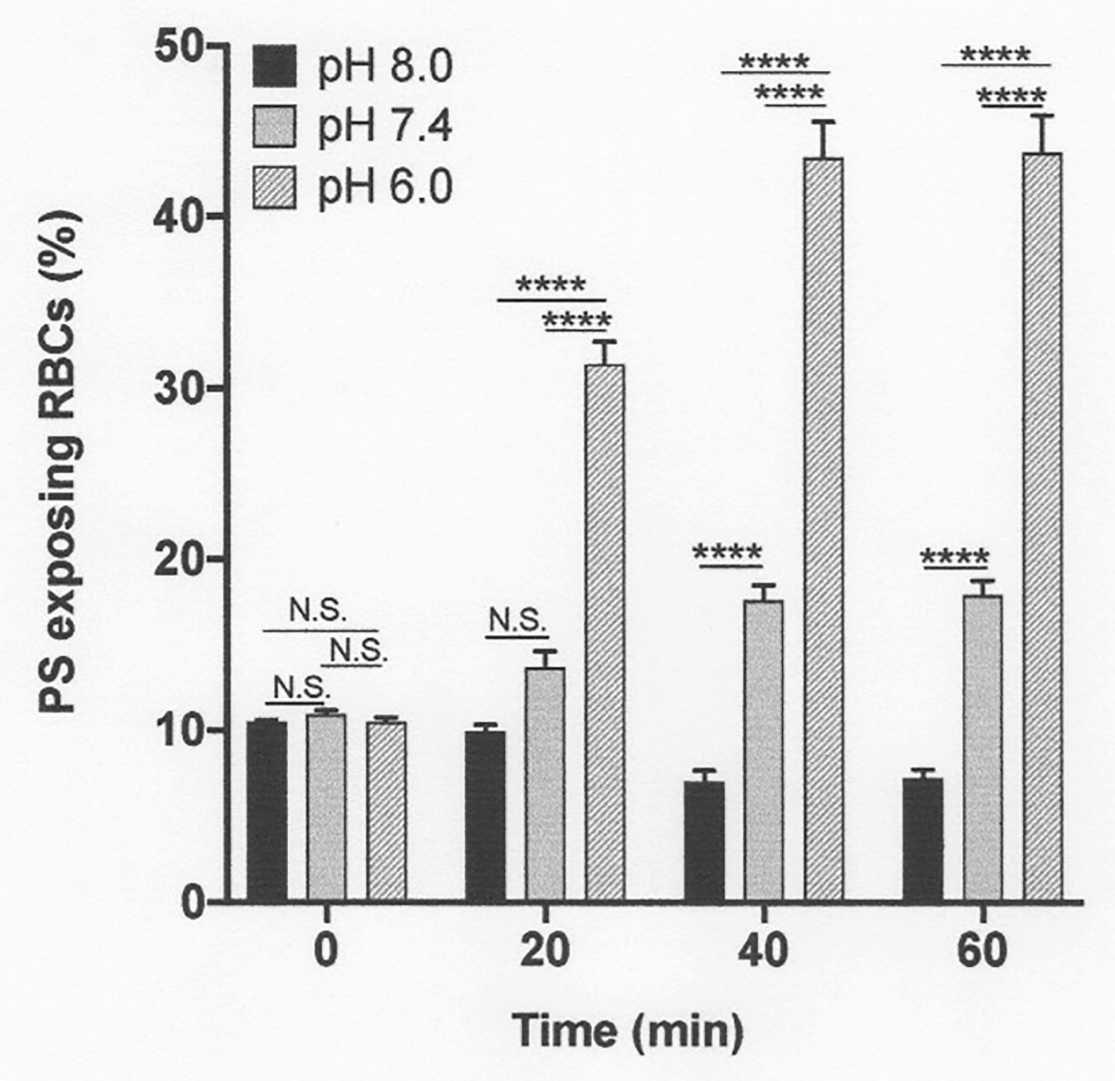
The effect pH on phosphatidylserine exposure in deoxygenated red cells from patients with SCA. Red cells (1% Hct) were incubated in Eschweiler tonometers at 37oC and an osmolality of 290 mOsm.kg-1 for up to 60 min under fully deoxygenated (0 mmHg oxygen) conditions. At the time intervals indicated, red cell aliquots were removed and PS exposure measured using LA-FITC, as described in the Methods. Histograms represent means ± SEM, *n* = 3. N.S: not significant; ^****^p < 0.0001.

**Figure 6:**
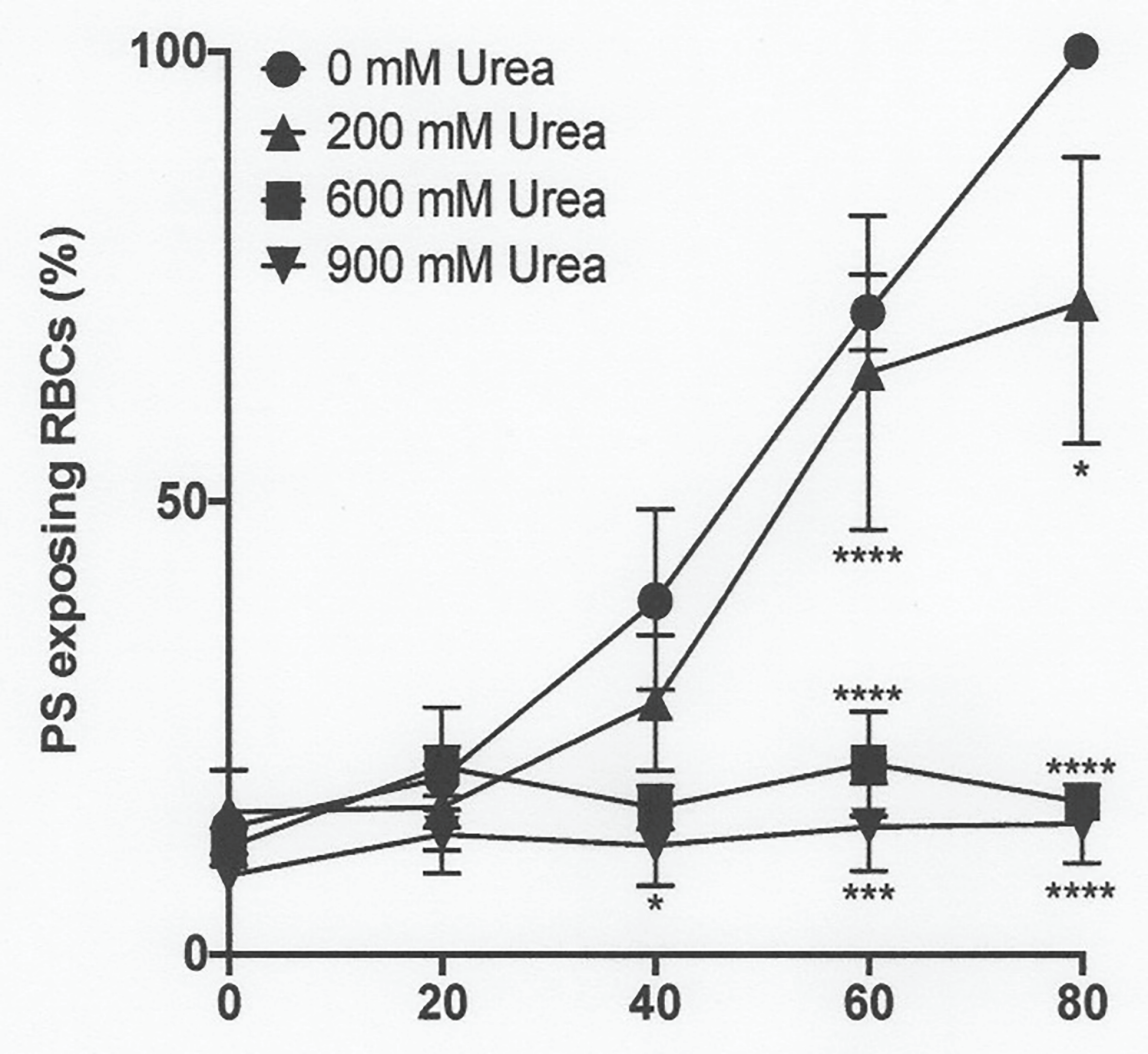
The effect urea on phosphatidylserine exposure in deoxygenated red cells from patients with SCA. Red cells (1% Hct) were incubated in Eschweiler tonometers at 37oC, pH 7.4 and an osmolality of 290 mOsm.kg-1 for up to 80 min under fully deoxygenated (0 mmHg oxygen) conditions in the absence of urea (0 mM urea) or at three different urea concentrations (200, 600, and 900 mM). At the time intervals indicated, red cell aliquots were removed and PS exposure measured using LA-FITC, as described in the Methods. Symbols represent means ± SEM, *n* = 3. ^*^p < 0.05; ^***^p < 0.001; and ^****^p < 0.0001, comparing red cells in the absence of urea with those in its presence.

**Figure 7:**
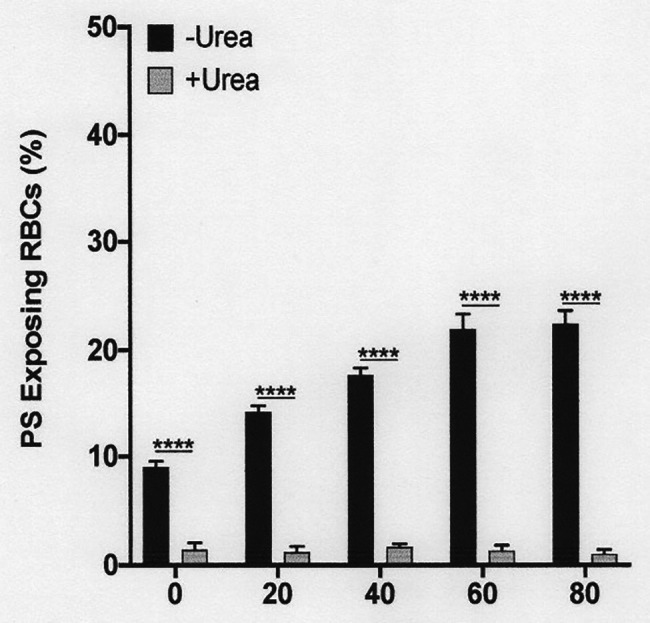
The effect urea on phosphatidylserine exposure at pH 7.0 in deoxygenated red cells from patients with SCA. Red cells (1% Hct) were incubated in Eschweiler tonometers at 37oC, pH 7.0 and an osmolality of 290 mOsm.kg-1 for up to 80 min under fully deoxygenated (0 mmHg oxygen) conditions in the absence of urea (-Urea) or in its presence (+Urea, 600 mM). At the time intervals indicated, red cell aliquots were removed and PS exposure measured using LA-FITC, as described in the Methods. Symbols represent means ± SEM, *n* = 3. ^****^p < 0.0001, comparing red cells in the absence of urea with those in its presence.

**Figure 8:**
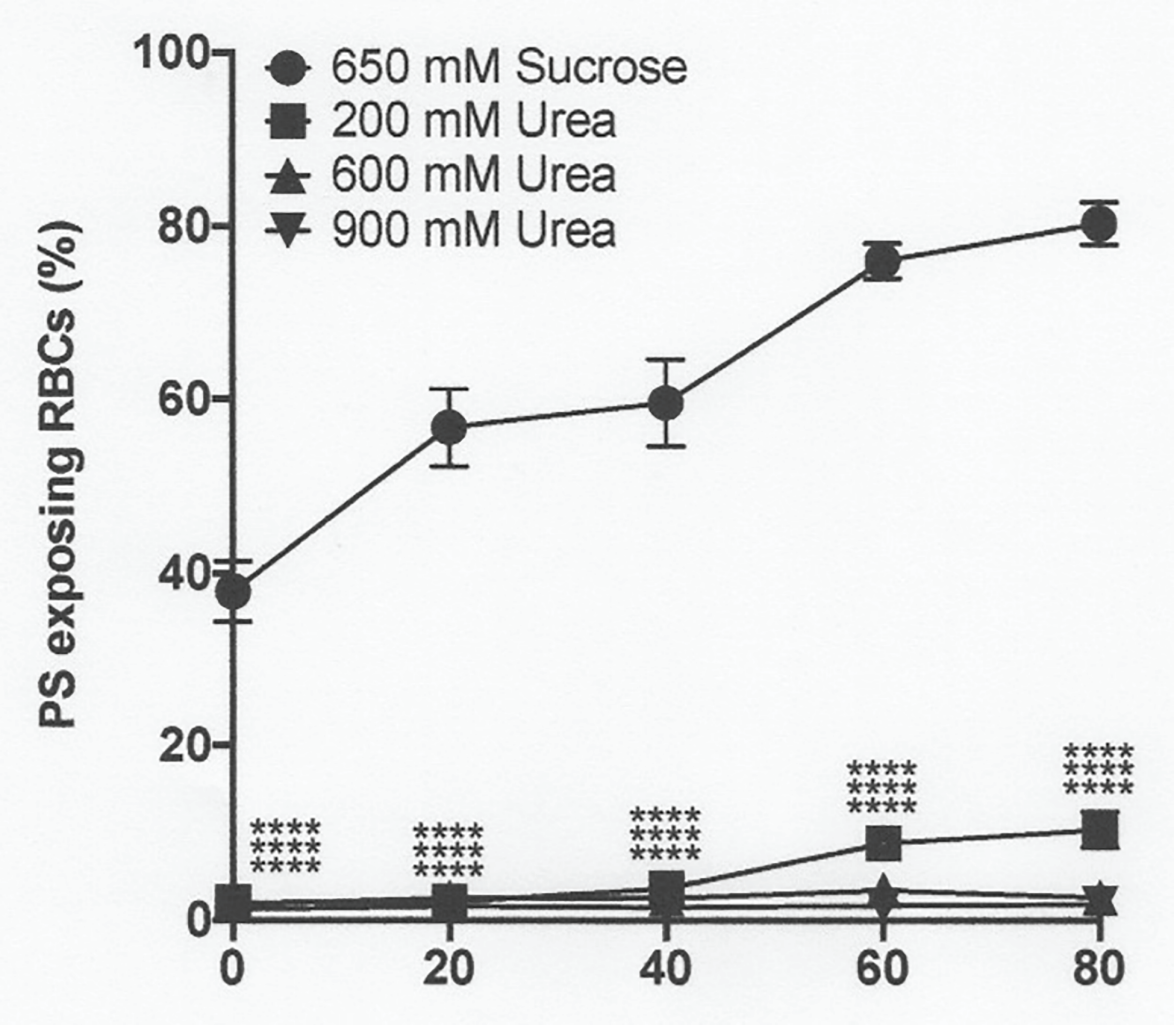
The effect urea and hypertonic sucrose on phosphatidylserine exposure in deoxygenated red cells from patients with SCA. Red cells (1% Hct) were incubated in Eschweiler tonometers at 37oC, pH 7.4 and at an osmolality of 940 mOsm.kg-1, through addition of hypertonic sucrose, for up to 80 min under fully deoxygenated conditions (0 mmHg oxygen) in the absence of urea (650 mM Sucrose) or at three different urea concentrations (200, 600, and 900 mM, all also with 650 mM Sucrose). At the time intervals indicated, red cell aliquots were removed and PS exposure measured using LA-FITC, as described in the Methods. Symbols represent means ± SEM, *n* = 3. ^****^p < 0.0001, comparing red cells in the absence of urea with those in its presence

Results are presented as means ± SEM for blood samples of *n* different SCA patients. Red cells under control conditions and exposed to different conditions (oxygen tension, pH, osmolality, or urea) were all paired. Statistical comparisons were made using two-tailed Student’s *t*-tests (Figure 1), two-way ANOVA (Figures 2, 4–8), or one-way ANOVA (Figure 3), as appropriate, and *p* < 0.05 was considered as significant.

**Figure 9:**
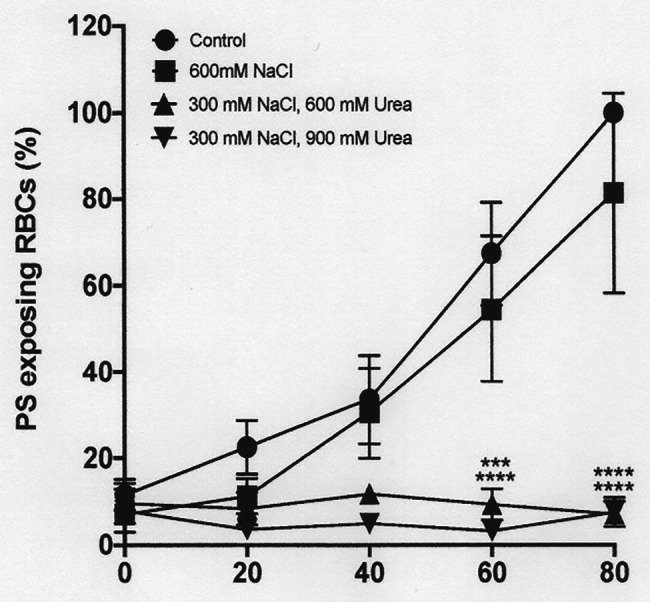
The effect urea and hypertonic NaCl on phosphatidylserine exposure in deoxygenated red cells from patients with SCA. Red cells (1% Hct) were incubated in Eschweiler tonometers at 37oC, pH 7.4 and an osmolality of 290 - 1200 mOsm.kg-1 (through addition of hypertonic NaCl) for up to 80 min under fully deoxygenated (0 mmHg oxygen) conditions in the absence of urea or at two different urea concentrations (600 and 900 mM). At the time intervals indicated, red cell aliquots were removed and PS exposure measured using LA-FITC, as described in the Methods. Symbols represent means ± SEM, *n* = 3. ^***^p < 0.01; and ^****^p < 0.0001., comparing red cells in the absence of urea at 290 mOsm.k

## Results

### The Effect of Hypoxia on Sickling and Phosphatidylserine Exposure in Red Cells From Patients With Sickle Cell Anaemia

Fully oxygenated red cells showed minimal sickling and PS exposure, with levels of both at less than 3%. As oxygen tension was lowered, both sickling and PS exposure increased. Sickling reached 57 ± 1% at an oxygen tension of 30 mmHg, rising to 80 ± 1% at 0 mmHg (Figure 1). Sickling was complete within about 20 min. PS exposure was also increased by deoxygenation but over a significantly slower time scale. In fully deoxygenated red cells, PS levels increased progressively with time such that levels were 5 ± 1, 10 ± 5, 12 ± 3, and 15 ± 5% after 20, 40, 60, and 80 min, respectively (Figure 2). These changes were significant at all time points after 20 min (values of *p* < 0.05, *p* < 0.01, and *p* < 0.001 after 40, 60, and 80 min, respectively). These findings confirm the well-established observations on the effect of deoxygenation on sickle cell behaviour (Blumenfeld et al., 1991; Weiss et al., 2012). In the following series of experiments, conditions were chosen to mimic those found in the renal medulla, investigating any effects on sickling and PS exposure. As results at 0 and 30 mmHg were similar, the former was chosen for most of the following hypoxic conditions.

### The Effect of pH on Sickling

The first set of experiments (shown in Figures 1, 2) was all carried out at pH 7.4. The next experiments investigated the effect of a range of pH values, from 8.0 to 6.5. Over these pHs, sickling remained minimal in fully oxygenated cells. In fully deoxygenated cells, after 15 min, sickling increased as pH was reduced, reaching 40 ± 1% at pH 7.4, 50 ± 1% at pH 7.0, and 60 ± 2% at pH 6.5 (Figure 3). As pH was increased from pH 7.4 to pH 8.0, sickling was modestly reduced by 13 ± 1%. In hypoxic conditions, metabolism will switch from aerobic to anaerobic with the likely accumulation of lactate – certainly in active muscle beds and also probably in the hypoxic renal medulla. The effect of addition of lactate was therefore also examined over this pH range. In the presence of 10 mM lactate, sickling in fully deoxygenated red cells increased significantly to 70 ± 1% (*p* < 0.05) at pH 7.4, 80 ± 1% (*p* < 0.05) at pH 7.0, and 90 ± 1% at pH 6.5 (all *p* < 0.05 cf. absence of lactate).

### The Effect of High Urea Levels on Sickling

In the renal medulla during antidiuresis, urea normally accumulates to high levels. The effect of addition of urea over a concentration range of 200–900 mM was therefore investigated, first on sickling induced by deoxygenation at normal pH (pH 7.4) and osmolality (290 mOsm.kg^−1^). At all concentrations of urea tested, sickling was reduced, albeit that this reduction was not significant at 200 mM. At the other urea concentrations (600 and 900 mM), statistical significance was reached (Figure 4).

### The Effect of pH on Phosphatidylserine Exposure

The subsequent series of experiments investigated the effect of conditions present in the renal medulla on PS exposure. First, the effects of pH changes were determined (Figure 5). In fully deoxygenated red cells, at pH 8.0, PS exposure was significantly reduced compared to that at pH 7.4, whilst at lower pH, pH 6.0, PS levels increased (Figure 5). These effects were also time dependent. For example, at pH 6.0, PS exposure increased progressively from 10 ± 1% at the start of incubation to 31 ± 3, 43 ± 4, and 43 ± 4% after 20, 40, and 60 min, respectively.

### The Effect of Urea and pH on Deoxygenation-Induced Phosphatidylserine Exposure

As noted above, in fully deoxygenated red cells, PS exposure increased progressively with time. The effect of urea was then examined. Under these conditions, lower urea concentrations (200 mM) had a modest inhibitory and insignificant effect, but higher concentrations (600 and 900 mM) essentially abrogated PS exposure, shown at pH 7.4 (Figure 6) and also for pH 7.0 (Figure 7). Similar findings were obtained at all other pHs, pHs 5.5–8.0 (data not shown). Further work focused on the effects of the higher urea concentrations tested.

### The Effect of Urea and Hypertonicity on Phosphatidylserine Exposure

Hypertonicity achieved through addition of sucrose also markedly increased PS, as observed previously in sickle cells by Lang et al. (2002, CBP). In a representative experiment, with the addition to saline of 650 mM sucrose, which increased osmolality from 290 to 940 mOsm.kg^−1^, PS in fully deoxygenated red cells increased from 11 ± 8% at the start of incubation to 39 ± 7, 71 ± 2, 81 ± 3, and 83 ± 1% after 20, 40, 60, and 80 min (all *p* < 0.05 cf. PS at time 0; Figure 8). Addition of NaCl (to an osmolality of 1,200 mOsm.kg^−1^), however, had minimal effect to the progressive increase in PS exposure during deoxygenation (Figure 9). In deoxygenated red cells, whether osmolality was increased through addition of sucrose (Figure 8) or NaCl (Figure 9), the presence of high levels of urea (600 or 900 mM) prevented any increase in PS exposure over the time course of the experiment.

## Discussion

The present findings show that the main conditions found in a healthy renal medulla during antidiuresis – namely hypoxia, acidosis, lactic acid accumulation, and hypertonicity – increased sickling and PS exposure in red cells from patients with SCA. High concentrations of urea markedly reduced these effects.

Sickle cell anaemia patients present with considerable morbidity and increased mortality, with many systems affected. The aetiology has been known for many decades (Bunn and Forget, 1986). The underlying event is the ability of the mutated form of adult haemoglobin, HbS, to aggregate upon deoxygenation, forming organised rigid polymers of sickle haemoglobin, which distort red cell morphology and result in numerous other changes to red cell behaviour. Notwithstanding the simple underlying cause, mechanisms of pathogenesis are multiple (Hebbel, 1991), so that, in many cases, the more direct causes of it is unclear exactly how particular complications arise.

Patients have a particularly high incidence of renal pathology, sometimes referred to as sickle cell nephropathy. Children initially show hyperperfusion, hyperfiterability, and glomerular hypertrophy, with subsequent reduced glomerular fitration rates (GFRs). In a substantial proportion of individuals, the disease progresses to chronic renal failure. Many older children and adults show evidence of fibrosis, sclerosis, and necrosis, with particular involvement of the renal medulla. A progressive decrease in ability to concentrate urea often occurs – hyposthenuria – and about a third goes on to require dialysis or renal transplant.

The sluggish blood flow coupled with a high metabolic activity at this site cause conditions within the renal medulla to be markedly hypoxic and acidotic. When patients retain a functional medulla, the medulla will also be hypertonic during antidiuresis with accumulation of salt (NaCl) and urea, with both reaching concentrations up to 600 mOsm.kg^−1^ (Roy et al., 1992). Total osmolality may reach about 1,200 mOsm.kg^−1^. All of these conditions would be expected to encourage HbS polymerisation and exacerbate sickling (Perillie and Epstein, 1963; Bookchin et al., 1976) with all the consequent deleterious changes, including PS exposure, adhesion to endothelial and other cells, and cohesion to themselves. These features could contribute to medullary damage by blocking blood vessels in this region with resultant ischaemia.

In this work, the conditions found in a functional renal medulla were mimicked to investigate their effect on sickle cell behaviour. Hypoxia, low pH, lactic acid, and hypertonicity were all found to increase sickling and PS exposure. Hypertonicity induced by sucrose, however, was much more effective than that elicited by high levels of NaCl. Urea at high concentrations levels was protective, particularly against PS exposure. We speculate that progressive loss of the ability to accumulate urea, which would occur as the renal medulla became progressively damaged would contribute to pathogenesis.

The effects on red cells of osmotic shock have been studied extensively by the group of Lang et al. (2002), although mainly on normal (HbA) red cells. They have shown that hypertonicity increases PS exposure and that this effect was exacerbated in sickle cells. In normal red cells, PS exposure induced by hypertonicity involves several pathways: it causes the opening of cation channels permeable to Ca^2+^ (Lang et al., 2004) and also stimulates sphingomyelinase with resulting increase in levels of ceramide, a compound associated with PS externalisation (Lang et al., 2004). Furthermore, hypertonicity also acts stimulates cyclooxygenase with production of PGE2, which acts *via* phospholipase A2 to activate sphingomyelinase (Lang et al., 2005). As for sickle cells, hypertonic NaCl was much less effective in causing PS exposure than the non-electrolyte sucrose (Lang et al., 2004), possibly through oxidative stress which also stimulates PS exposure (Duranton et al., 2002; Hannemann et al., 2018). It will be interesting to investigate the involvement of these mechanisms in sickle cells.

Lang’s group also examined the protective effect of urea. In normal red cells and platelets, urea was also seen to reduce PS exposure induced by hyperosmotic shock (Lang et al., 2004; Gatidis et al., 2010). Urea appeared to act by inhibiting sphingomyelinase, reducing the synthesis of ceramide, and was thus ineffective in preventing PS exposure in response to added ceramide (Lang et al., 2004). Furthermore, urea did not block, but rather activated, the red cell cation conductance, indicating that this channel is not the main mechanism by which hyperosmotic shock results in PS exposure pointing to a pre-eminent contribution to ceramide (Lang et al., 2004, 2010).

Urea also probably affects the hydrophobic bonds within the HbS molecules that result in polymerisation (Nalbandian et al., 1972; May and Huehns, 1975). As such, it has been proposed as a possible treatment for SCA patients (McCurdy and Mahmood, 1971). In reality, however, it is likely that the concentrations required would be excessively high, near molar levels. Because of this, other potential reagents, such as cyanate, which prevent polymerisation at much lower (low mM) concentrations have been suggested (Cerami and Manning, 1971) although it has been shown to be ineffective as a clinical therapy for SCD.

Finally, a number of manoeuvres considered here, notably pH and urea, will also alter the permeability of the red cell membrane acting *via* transport systems, which include stimulation of the KCl cotransporter (KCC) and effects on P_sickle_ (Gibson and Ellory, 2002, 2003). Urea also stimulates KCC in red cells from other species (including dog, sheep, and horse) – see Gibson and Ellory (2003). Such changes in membrane permeability may therefore also contribute to effects on sickling and PS exposure.

In conclusion, the hypoxic, acidic, and hypertonic conditions in the renal medulla act to increase sickling and PS exposure in red cells from SCA patients, an effect markedly reduced by high concentrations of urea.

## Data Availability Statement

The raw data supporting the conclusions of this article will be made available by the authors, without undue reservation.

## Ethics Statement

The studies involving human participants were reviewed and approved by National Research Ethics Committee (reference 16/LO/1309). Written informed consent to participate in this study was provided by the participants’ legal guardian/next of kin.

## Author Contributions

JG and DR planned the research. Experiments were carried out by DL, RW, and AH. DR, JB, JG, RW, and DL wrote the manuscript. All authors contributed to the article and approved the submitted version.

### Conflict of Interest

The authors declare that the research was conducted in the absence of any commercial or financial relationships that could be construed as a potential conflict of interest.
